# Development of Blends to Improve Flexibility of Biodegradable Polymers

**DOI:** 10.3390/polym14235223

**Published:** 2022-12-01

**Authors:** María Jordá-Reolid, Ana Ibáñez-García, Linda Catani, Asunción Martínez-García

**Affiliations:** 1 Innovative Materials and Manufacturing Area, Technological Institute for Children’s Products and Leisure, 03440 Ibi, Spain; 2 Department of Biological, Geological and Environmental Science—BiGeA, Alma Mater Studiorum University of Bologna—Campus of Ravenna, 48121 Ravenna, Italy

**Keywords:** compatibility, blends, biopolymers

## Abstract

In this study, binary blends of biodegradable polymers were prepared to improve the ductile properties of those that have a more rigid and/or brittle behaviour. Specifically, PLA, PHA and TPS were blended with different amounts of PBS with the objective of reducing the stiffness and brittleness of the three polymers. The compatibility of the blends and their resulting mechanical properties were studied. The flexibility of the blends increased with the addition of PBS; however, a limited compatibility was achieved, leading to a low impact resistance improvement. For this reason, other blend options with an EVA-based material were studied, increasing the impact resistance and flexibility of the PLA material in this case.

## 1. Introduction

In recent decades, the importance that the sustainability and care of the environment have acquired has influenced all the stages in the production of products, including design, manufacturing, use, end-of-life, and transportation. In this process, an aspect worth considering is the choice of material out of which to manufacture a product, and in this respect, plastics are of great importance, since they have very advantageous properties (lightness, durability, toughness, relatively low brittleness, low stiffness, and good gas barrier and humidity barrier) for their use in a wide variety of products [1,2,3,4].

However, being such a widely used material, the use of plastic has resulted in a large amount of waste (over 6.3 billion tonnes of plastic waste have been generated worldwide [5]), which has, on many occasions, not been adequately treated through recycling, therefore causing environmental problems. For instance, substantial amounts of plastic are deposited in landfills that end up being incinerated or transfered to natural environments, such as forests or seas, thus causing problems such as seas of plastic and the presence of microplastic in water, food, and the environment (air) [6]. These issues have caused concern in the population due to both environmental pollution and potential health problems. Furthermore, the main origin of conventional plastic is oil, a non-renewable source that is in the spotlight for being unsustainable [5,6,7,8].

Research of biopolymers is extremely important, since, in some cases, their properties do not resemble the properties of conventional polymers. Chemically, some biopolymers have different structures and consequently different properties and behaviours than conventional polymers. This is the case for both biodegradable and not-biobased biopolymers. These differences make it difficult to manufacture some products with these polymers because they must be processed at different temperatures and the properties of the final parts are not the same as when conventional polymers are used. This is why some adaptations must be made in the processing of biodegradable polymers to improve their properties. In contrast, biobased polymers with the same chemical structures as conventional polymers, such as bioPE, bioPP, and bioPET, also share the same mechanical and thermal behaviours. These biopolymers differ in the origin of the components out of which they are synthesised, as they come from renewable sources instead of oil [1,4,9,10,11,12].

Previous studies have shown the viability of injection moulding, blow moulding or rotational moulding processes using formulations based on polycaprolactone (PCL), polylactic acid (PLA), thermoplastic starch (TPS), or polybutylene succinate (PBS), as well as blends of these with natural fillers of the lignocellulosic type. Studies have also been carried out on the improvement of plastics’ mechanical properties, for example via techniques that use blends and biodegradable materials [13,14] and techniques that use natural additives, such as dyes, antimicrobials and flame-retardants [15,16,17,18].

One of the properties that makes it difficult to use several biodegradable polymers for specific applications is rigidity, as in the case for polylactic acid (PLA), some polyhydroxyalkanoates (PHAs), and TPS (thermoplastic starch) [8,19,20,21]. This is why the decrease in rigidity of these three families of biodegradable polymers has been studied with different strategies, such as the use of plasticisers, that help to increase the flexibility of these polymers and facilitate their processing in transformation methods, such as extrusion and injection moulding. It is preferred that these plasticisers are of renewable origin and biodegradable so that they do not hinder the degradability of the final polymer, which is why some vegetable oils, such as corn oil, soybean oil, and linseed oil, are used [22,23,24,25,26]. Another strategy is to develop blends of these polymers with other, more flexible ones such as PBS or bioPBS (PBS is a biodegradable polymer of petrochemical origin and bioPBS is this same polymer whose synthetic components are renewable) that have a low Young’s modulus ranging from 320 MPa to 645 MPa [1,27].

For these reasons, in this work, binary blends of biopolymers were developed to improve the ductile properties of those that have a more rigid behaviour. Specifically, polylactic acid (PLA), polyhydroxyalkanoate (PHA), and thermoplastic starch (TPS) were selected to be blended with polybutylene succinate (PBS) with the objective of reducing the stiffness and brittleness of the three polymers. The compatibility and resulting mechanical properties of the blends were studied. Additionally, the use of blends of PLA with an additive based on ethylene-vinyl acetate (EVA) was investigated as another way of increasing the flexibility of PLA.

## 2. Materials and Methods

### 2.1. Materials

The materials used in this study were PLA LL652 and PHA PH070, both supplied by Ercros, Barcelona, Spain; TPS Mater-Bi EI51N0, supplied by Novamont (Novara, Italy); and biobased PBS, supplied by PTTMCC Biochem (Bangkok, Thailand). Additionally, the ethylene-vinyl acetate-based additive was Vinnex 2504, supplied by Wacker (Munich, Germany).

### 2.2. Blends Preparation

PLA/PBS, PHA/PBS and TPS/PBS blends were initially prepared by mixing 40 g of each blend in a Brabender GmbH & Co. KG (Duisburg, Germany) plastograph at a spindle speed of 50 rpm for 3 min. Temperatures of 200 °C, 190 °C, and 160 °C were used for the PLA, TPS, and PHA blends, respectively. Different percentages of PBS were added (0 wt%, 20 wt%, 40 wt%, 60 wt%, 80 wt%, and 100 wt%), and the compatibility of the blends was studied with differential scanning calorimetry (DSC) and scanning electron microscopy (SEM). 

To mechanically characterise the blends, standard test samples were prepared via the extrusion–compounding of the three biopolymers with 20 wt% and 40 wt% of PBS followed by injection moulding. The extrusion temperatures for PLA along the different sections of the screw (from nozzle to feeder) were 195 °C (nozzle), 195 °C, 195 °C, 190 °C, 190 °C, 190 °C, and 40 °C (feeder); those for TPS were 205 °C (nozzle), 205 °C, 205 °C, 200 °C, 200 °C, 200 °C, and 40 °C (feeder); and those for the PHA were 160 °C (nozzle), 160 °C, 150 °C, 150 °C, 140 °C, 140 °C, and 40 °C (feeder). The injection conditions for each used material are shown in Table 1.

The preparation of the PLA blends with the vinyl acetate/ethylene-based additive was carried out via an extrusion–compounding process. As the additive was in a powder form, extrusion–compounding was carried out to introduce the powder additive to the polymeric matrix with 20 wt% of additive. The extrusion temperatures were 195 °C (nozzle), 195 °C, 195 °C, 190 °C, 190 °C, 190 °C, and 40 °C (feeder). Then, a second extrusion process was carried out to obtain diluted blends from the 20 wt% blend in order to obtain PLA/additive blends at 15, 10 and 5 wt% additive.

Once the blends were obtained, they were injected to obtain standardised tensile specimens. The injection conditions are summarised in Table 2.

### 2.3. Characterisation

The obtained PLA/PBS blends were characterised by differential scanning calorimetry (DSC) and scanning electron microscopy (SEM) to study the compatibility of the polymers. Mechanical characterisation was carried out by tensile, flexure, and Charpy impact tests.

DSC tests were carried out on a Q200 calorimeter from TA Instruments (New Castle, DE, USA). A dynamic thermal program was scheduled in three different stages using standard aluminium crucibles. The first heating from 30 °C to 200 °C was followed by a cooling down to −90 °C and a second heating up to 350 °C. The heating rate was set to 20 °C·min^−1^, and the cooling rate was set to 5 °C with a constant nitrogen flow rate of 50 mL min^−1^.

The morphological characterisation of the Charpy impact rupture was carried out through SEM by using a JEOL SEM J840 (Akishima, Tokyo, Japan) microscope with an electron acceleration voltage of 15 kV. The morphology was additionally studied by solving the minor phase to observe the holes left by PBS in the matrix of PLA, PHA and TPS. 2-methyl-sulfoxide was used as the solvent of PBS; the samples were immersed in it for 20 min and subsequently dried for 60 min [28,29,30]. Then, morphology was observed using a SCIOS 2 FIB-SEM electronic microscope with an electron acceleration voltage of 3 kV.

The tensile properties of the blends were characterised by testing dog bone samples with a Zwick Roell INSTRON 6025 universal testing machine (Ulm, Germany), as recommended by ISO 527-1:2012 standard. All tests were run at a crosshead speed of 10 mm·min^−1^ using a 10 kN load cell. The impact strength was also studied with injection-moulded rectangular samples with dimensions of 80 × 10 × 4 mm^3^ in a Charpy pendulum Instron (Cerdanyola Del Vallès, Barcelona, Spain) following the specifications of ISO 179-1:2010. Notched specimens and a 1 joule hammer were used for the PBS blends, and unnotched specimens and a 5 joule hammer were used for the PLA/additive blends.

## 3. Results and Discussion

### 3.1. Compatibility Study

The compatibility of the components of each mixture was studied using two complementary techniques. First, it was studied with differential scanning calorimetry (DSC), paying special attention to the thermal transitions of each component (glass transition and melting temperatures of the material) and the crystallinities of the material after an initial heating treatment carried out to eliminate the thermal history in the stages of material processing (extrusion, pelletizing and injection). Observing the changes in transition temperatures is important for a study of compatibility, since an approximation of the glass transitions or melting point of the two components of blends supposes an interaction between components, as described in the literature [20,21].

Additionally, scanning electron microscopy (SEM) was used. This technique allowed to observe the different phases of the mixtures, as well as their interactions. In the case of a good interaction, only a homogeneous phase was observed. Otherwise, a heterogeneity of different phases was observed.

**Figure 1:**
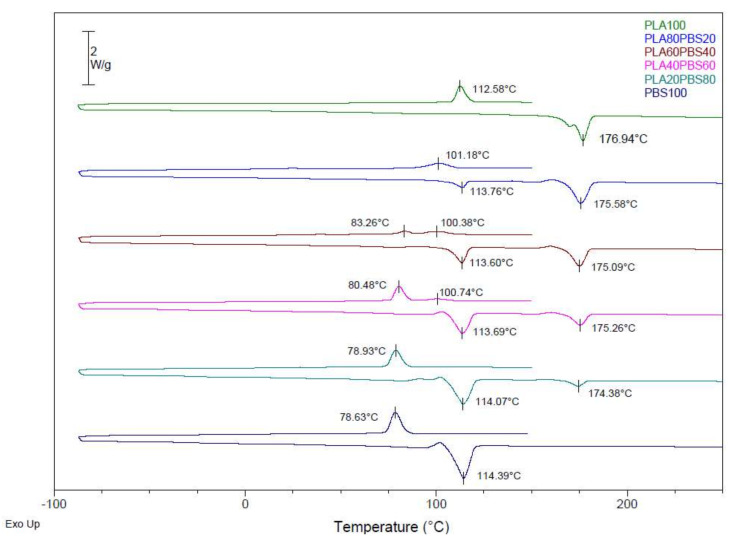
DSC curves of PLA/PBS blends.

**Figure 2:**
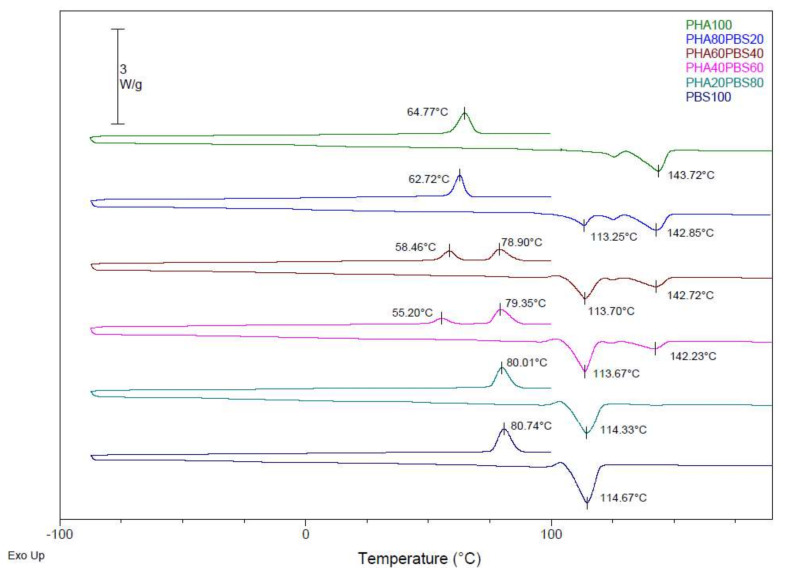
DSC curves of PHA/PBS blends.

**Figure 3:**
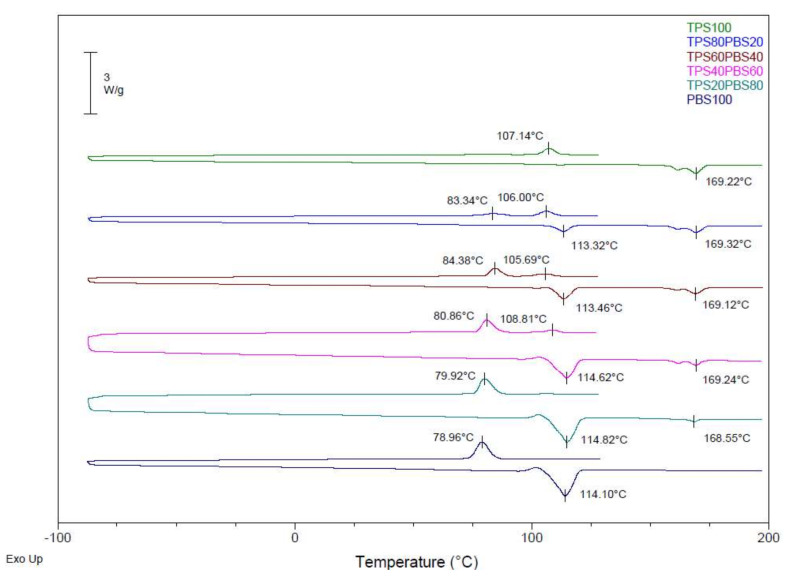
DSC curves of PHA/PBS blends.

The calorimetric curves obtained for the blends of PLA, PHA, and TPS with PBS are shown in Figure 1, Figure 2 and Figure 3. A first heating treatment was carried out to erase the thermal history of the material, then a cooling cycle, and a second heating treatment were performed to assess the thermal transitions of each component of the mixtures. The maximum degree of crystallinity was calculated for each component of the blends (see Equation (1)) by comparing the melt enthalpy (∆H_m_) with the corresponding melt enthalpy of a theoretical 100% crystalline polymer ($H_{m}^{0}$ for PLA = 93.7 J·g ^−1^ [31], $H_{m}^{0}$ for PHA = 146 J·g ^−1^ [32], and 110.3 J·g ^−1^ for PBS [33]) while considering the weight fraction of each polymer in the blend (ω).

(1) $$\%\chi =\frac{\Delta H_{m}}{{\Delta H}_{m}^{0}\cdot \omega }$$

Figure 1 shows the thermograms of the PLA and PBS blends. In the calorimetric curves, the melting temperatures and crystallisation temperatures for each component can be observed according to the amount of PLA and PBS in each mixture. In addition, the melting enthalpies and the degree of crystallinity of each component for each formulation were calculated. The temperature, enthalpy, and crystallinity values are included in Table 3.

The melting temperatures of the as-received PLA and PBS were 176.94 °C and 114.39 °C, respectively. With the addition of PBS in different percentages (from 20 wt% to 80 wt%), the two aforementioned melting points were still observed, indicating that no complete compatibility of the materials was produced. These results denote a lack of chemical interaction between both polymers. The melting point of the PLA was slightly shifted to the left, up to 174.38 °C in the 20/80 blend, and the melting point of the PBS was also shifted to the left. The PLA20PBS80 formulation was the blend with the closest melting points of its components (T_m1_ and T_m2_). Despite the different formulations, there were no significant differences in melting temperatures when blending PLA with PBS.

Regarding the crystallinity of both components (PLA and PBS), a decrease in crystallinity was observed depending on the amount of PBS. When the amount of PBS increased and the amount of PLA decreased in the formulation, the crystallinity of PLA was lower. On the contrary, the percentage of crystallinity of the PBS did not show a linear behaviour according to the percentage of each component in the formulation. A slight decrease was observed with the addition of PBS up to 60 wt%. With this amount of PBS, the crystallinity increased up to 53.64% when PBS was not blended with PLA. Therefore, it can be concluded that the crystallinity of PBS was not affected by the content of PLA in the studied formulations.

Figure 2 shows thermograms of the PHA and PBS blends. In the calorimetric curves, the melting temperatures and crystallisation temperatures for each component can be observed according to the amount of PHA and PBS in each blend. In addition, the melting enthalpies and the degree of crystallinity of each component for each formulation were calculated. The temperature, enthalpy, and crystallinity values are shown in Table 4.

The melting temperature of the as-received PHA was 143.72 °C, and the melting temperature of the as-received PBS was 114.67 °C. With the addition of the PBS to the PHA, the melting temperature of the PHA was slightly modified; variations were about 1 °C. The melting point of the PBS was also shifted a bit to the left, with PHA60PBS40 being the blend with the closest melting points. Therefore, as both melting points were maintained, these small variations in melting temperatures did not show a good interaction between both polymers.

It is observed that the crystallinity of the PBS in the PHA/PBS blends was higher than the crystallinity of the PBS in the PLA blends, reaching 66.68% of crystallinity with the PHA80PBS20 formulation compared with 50.5% of crystallinity in the PLA80PBS20 formulation. This crystallinity of the formulation was reduced with increases in the content of PBS up to 47.65% when the formulation contained 80 wt% of PBS. In the case of the PHA, its crystallinity (31.63%) was lower than that of the PLA (50.88%). The crystallinity of the PHA was decreased with increasing percentages of PBS, being 5.40% with a PBS content of 80 wt%.

Figure 3 shows the thermograms of the TPS/PBS blends. In the calorimetric curves, the melting and crystallisation temperatures for each component can be observed according to the amount of PLA and PBS in the blends. In addition, the melting enthalpies and the degree of crystallinity of the PBS for each formulation were calculated. In the case of TPS, it was not possible to calculate the degree of crystallinity of the TPS because reference melting enthalpy values for 100% crystalline TPS cannot be found in the literature. Temperature, enthalpy and crystallinity values are shown in Table 5.

The melting temperature of the as-received TPS and PBS were 169.20 °C and 114.06 °C, respectively. With the addition of the PBS, the melting temperature of the TPS in the blends was not affected; though it suffered from some variations, they were only of a few tenths of a degree. The temperature of the PBS somewhat shifted to the left when it was in a lower proportion, so that at low percentages of PBS in the blends, the melting temperatures of the PBS and TPS separated. As the amount of PBS in the blends increased, the melting temperature of the PBS slightly approached the melting temperature of the TPS. Therefore, it can be concluded that there was a lack of chemical interaction or compatibility between both materials.

The crystallinity of the PBS in these formulations did not present linear variations. The crystallinity value of the TPS80PBS20 formulation was 56.80%, and it decreased to 49.27% when the PBS content increased up to 40 wt%, increased to 65.31% at a PBS percentage of 60 wt%, and then decreased again to 53.64 wt% for virgin PBS.

**Figure 4:**
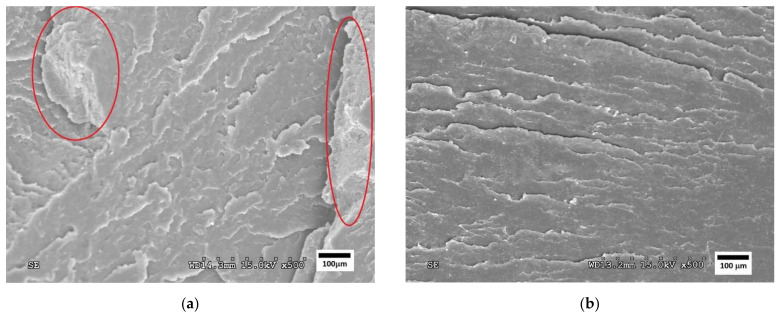
SEM micrographs of fractured surfaces at 500× of (**a**) PLA80PBS20 and (**b**) PLA60PBS40 blends.

**Figure 5:**
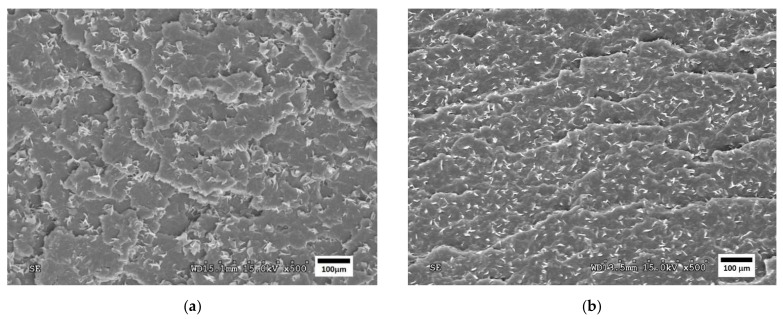
SEM micrographs of fractured surfaces at 500× of (**a**) PHA80PBS20 and (**b**) PHA60PBS40 blends.

**Figure 6:**
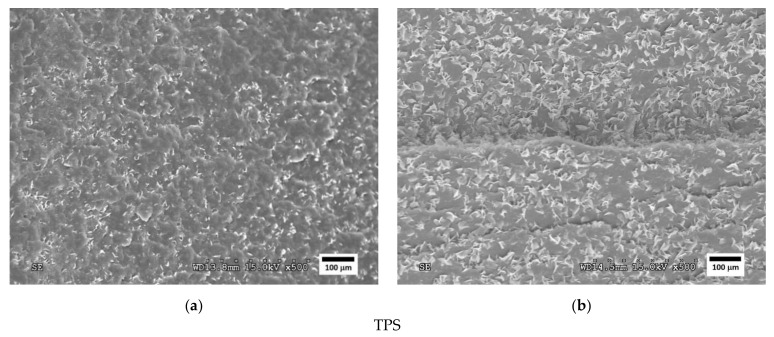
SEM micrographs of fractured surfaces at 500× of (**a**) TPS80PBS20 and (**b**) TPS60PBS40 blends.

Figure 4, Figure 5 and Figure 6 show SEM images of the fractured surfaces of specimens after the Charpy impact test. These fractures were observed at 500× for the PLA, PHA and TPS blends, respectively.

The micrographs of the PLA/PBS formulation showed a fragile fracture since many edges could be seen where the material was delaminated, with a very brittle break. On these edges, important roughness was observed, in particular for the PLA80PBS20 formulation (Figure 4a). On the right side of this image, an edge can be observed at a higher plane to the rest of the fracture in which the roughness coincides with some holes that remain on the surface of the break. This shows a lack of homogeneity between both materials, which could have caused the brittleness of the material.

In addition, the PLA60PBS40 formulation (Figure 4b) showed a brittle behaviour. However, in this case, the fracture showed less marked delamination, so it could be stated that this second formulation presented a greater ductile behaviour than the PLA80PBS20, due to its higher PBS content.

The PHA/PBS micrographs in Figure 5 show some small layers in the fracture lines that were delaminated from the PHA matrix. These were caused by the more ductile behaviour of the PHA than the studied PLA. The micrograph of PHA80PBS20 (Figure 5a) shows different edges at different parallel planes, as well as a high amount of fracture lines with a brittle aspect. However, the microscopy of the PHA60PBS40 formulation (Figure 5b) revealed a lower number of fracture lines and a higher number of layers than PHA80PBS20. In addition, the fracture lines showed greater curvatures without appearing so brittle in the PHA80PBS20 formulation. For these reasons, it can be deduced that the observed small fragments resulted from the presence of PBS, and they became more abundant with increases in PBS content. Additionally, the increase in PBS provided ductility to the material, making the behaviour of the fractures in the PHA60PBS40 formulation more similar to ductile fractures.

In micrographs of the TPS/PBS formulations (Figure 6), a similar behaviour to the PHA/PBS can be observed, though in this case, the material showed a more ductile behaviour since a greater amount of small layers was observed at the edges of the fractures. This phenomenon was observed in both formulations, but the amount of small layers in TPS600PBS40 (Figure 6b) was higher than the amount in TPS80PBS20 (Figure 6a).

Therefore, the SEM analysis confirmed the lack of compatibility between the three polymers and the PBS revealed by the DSC analysis. The micrographs show phase separation in the case of the PLA/PBS formulations, as can be seen in the appearance of small sheets that are separated from the matrix and create a “delamination” in both the PHA/PBS and TPS/PBS formulations.

In order to study the morphology and to confirm the homogeneity and lack of compatibility of the blends, the etching of the minor phase was carried out using a specific solvent for the PBS (DMSO).

**Figure 7:**
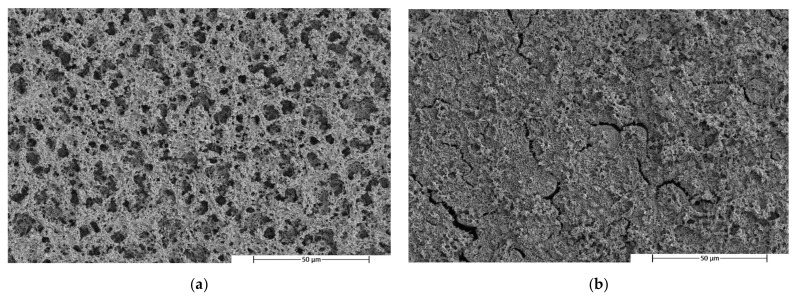
SEM micrographs of fractured surfaces after minor phase solving at 2500× of (**a**) PLA80PBS20 and (**b**) PLA60PBS40 blends.

**Figure 8:**
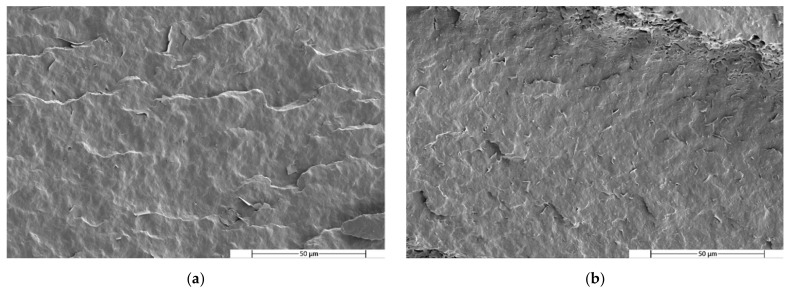
SEM micrographs of fractured surfaces after minor phase solving at 2500× of (**a**) PHA80PBS20 and (**b**) PHA60PBS40 blends.

**Figure 9:**
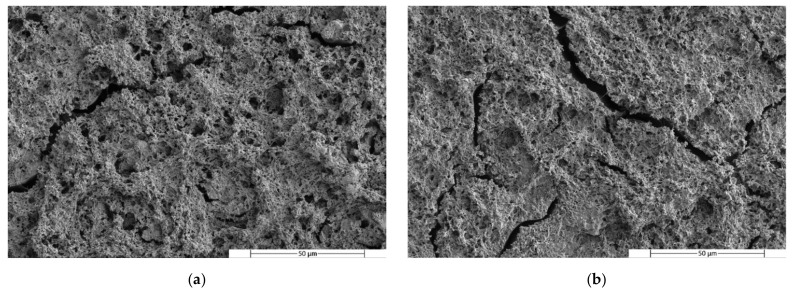
SEM micrographs of fractured surfaces after minor phase solving at 2500× of (**a**) TPS80PBS20 and (**b**) TPS60PBS40 blends.

Figure 7, Figure 8 and Figure 9 show SEM images of the PLA, PHA and TPS blend surfaces after minor phase solving at 25,000×, respectively.

The micrographs of the PLA/PBS blends taken after PBS dissolution show some holes at the PLA matrix. It can be observed that these holes were homogeneously distributed in the matrix, indicating that the PBS was homogeneously distributed throughout the matrix. On the other hand, the presence of the holes means that the material had two phases that did not correctly interact with each other, presenting a low compatibility. When the percentage of PBS in the blend increased from 20% (Figure 7) to 40% (Figure 7b), variations in the surface micrograph cracks could be observed in different areas of the surface and the holes became smaller.

The micrographs corresponding to the PHA/PBS (Figure 8) blends looked very different than the micrographs corresponding to the previous PLA/PBS mixtures. In this case, gaps generated by the elimination of PBS were not observed. On the contrary, surfaces with undulations could be observed. These surfaces had quite brittle edges, which indicates the somewhat brittle behaviour of the material. The absence of holes may have been due to the complete dissolution of the surface.

A similar behaviour to that of the PLA/PBS blends was observed in the micrographs of the TPS/PBS blends. After etching with DMSO, small holes left by the PBS in the TPS matrix were observed. In this case, these holes had a similar distribution in both blend formulations. However, some larger holes can be observed in the first micrograph (Figure 9a), as in the micrograph of the PLA80PBS20 blend (Figure 7a). In addition, the appearance of cracks was also observed in both micrographs.

### 3.2. Mechanical Properties

The mechanical properties of the PLA/PBS, TPS/PBS and PHA/PBS blends were studied. Table 6 shows the results of the tensile and Charpy impact tests.

With the addition of PBS to the PLA, TPS and PHA, a decrease in Young’s modulus (E_t_) was observed. Additionally, the maximum tensile stress (σ_m_) decreased, except for the PHA blends because the virgin material had a maximum stress value of 25.6 MPa, which was lower than that of the virgin PBS (34.6 MPa). The decrease of these two parameters denoted a drop in the stiffness of the material due to the ductility contribution of the PBS, as also demonstrated the SEM images. On the other hand, with the addition of PBS, an important increase in the elongation (ε_b_) of all the blends was observed. In the case of PLA, this represented an increase of 236% of the initial elongation value (from a value of 2.5% up to 8.45%). However, in the other two studied blends, this increase was higher, almost 700% in the TPS60/PBS40 blend and 460% in the PHA60/PBS40 blend. This effect again demonstrated an increase in ductile properties with the addition of PBS.

On the contrary, the results of the impact test were unexpected, with increases in the ductile properties and, therefore, the impact energy absorption. This occurred in both blends with PLA and TPS, in which the addition of PBS decreased the impact resistance. Only in the case of the PHA blends did the impact energy increase with the addition of PBS. This decrease in impact energy can be explained by the lack of compatibility between the PLA matrix and TPS with PBS, causing the lack of homogeneity in the blends that hindered the transmission of impact forces throughout the parts and aiding breakage in those areas between the two phases of the heterogeneous blend.

Table 7 shows the results of the flexural tests, which corroborate the conclusions obtained from the analysis of the results of the tensile and impact tests. The flexural modulus decreased with the addition of PBS to the blends. Additionally, the maximum tensile stress increased was mainly affected with PBS addition at 40 wt%. As in the tensile test, the strain, measured as the maximum stress, increased for the three materials with the addition of PBS.

As a conclusion of the mechanical characterisation, blending rigid materials with other, more flexible materials enables a more ductile material to be obtained, although it is important to study the compatibility and affinity of the created material; it may be necessary to use compatibilizers.

The limited compatibility and, therefore, the low impact resistance improvement of the previously developed blends led to the study of other blend options. Specifically, PLA blends with an EVA-based material were studied. Mechanical characterisation was carried out using tensile and impact tests.

Table 8 shows the tensile results of the PLA blends with the EVA-based additive added in different amounts (0, 5, 10, 15 and 20 wt%). It can be appreciated that rigidity considerably decreased with the additive content, as indicated by the Young’ modulus. The maximum tensile stress also decreased, whereas elongation at break increased up to more than 900% of the initial elongation value.

**Figure 10:**
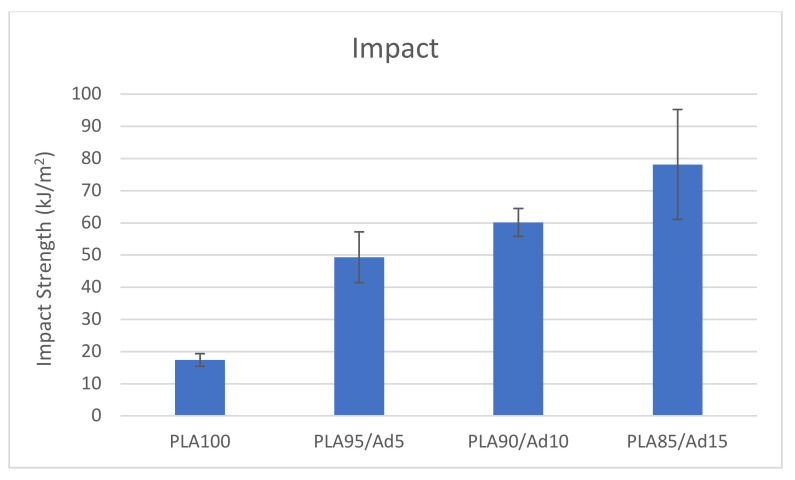
Graphic representation of impact test results of PLA/additive blends.

The results of the impact test are graphically shown in Figure 10, where a significant increase in impact strength can be observed as the additive content increased. A very ductile material that was capable of homogeneously distributing the energy of the impact throughout the piece before breakage was obtained. This can be attributed to the good chemical affinity between the PLA and the EVA-based additive.

The results of the impact test for the blend with 20% of additive are not shown in the graph since when the test was performed with unnotched specimens, the material did not break with the used pendulum (5 J). This means that its impact resistance was greater than the rest of the studied blends. This behaviour was expected, since the function of the additive was to provide ductility to the PLA.

Therefore, appropriate impact resistance and flexibility values of PLA were achieved with these blends.

## 4. Conclusions

In the present work, it was observed that the addition of PBS to more rigid biopolymers such as PLA, TPS and PHA was able to improve its flexibility. However, a lack of compatibility between the PBS and the studied polymers that caused a decrease in the impact resistance of the blends was observed. Despite the lack of compatibility with the PBS, the ductile properties of the three polymers (PLA, PHA and TPS) were increased. Decreases in the tensile modulus, flexural modulus, and maximum tensile stress and an increase in deformation were observed as expected. Additionally, the Charpy impact of PHA was improved following the addition of PBS but decreased in the TPS blends and did not undergo significant variations in the PLA blends.

On the other hand, with the addition of a commercial additive based on a vinyl acetate-ethylene copolymer, the ductility of the resulting PLA blends was notably increased, balancing the stiffness of the PLA itself. These blends were also found to be highly resistant to impact tests compared with the impact behaviour of PLA. These results support PLA’s use in many types of toy products, which, in accordance with safety regulations, must not break and generate small pieces. A disadvantage of using the aforementioned commercial additive is that it is not biodegradable, so it reduces the degradability of PLA. This should be taken into account when communicating the real biodegradable content of manufactured products.

## Figures and Tables

**Table 1:** Injection moulding conditions of binary blends.

Conditions	PLA Blends	TPS Blends	PHA Blends
Injection temperature (°C)	210–210–200–190–50	220–220–200–180–50	160–160–150–140–50
Mould temperature (°C)	35	30	35
Speed (cm^3^/s)	60	60	30
Compaction time (s)	15	20	30
Compaction pressure (bar)	450	450	350
Cooling time (s)	60	40	80
Loading speed (rpm)	100	100	100

**Table 2:** Injection moulding conditions of PLA/vinyl acetate blends.

Conditions	PLA/Additive
Injection temperature (°C)	200–200–190–180–50
Mould temperature (°C)	35
Speed (cm^3^/s)	60
Compaction time (s)	10
Compaction pressure (bar)	550
Cooling time (s)	40
Loading speed (rpm)	100

**Table 3:** Transition temperature and crystallinity of PLA/PBS blends.

Formulations	T_m1_	$\Delta H_{m1}^{0}$	T_m2_	$\Delta H_{m2}^{0}$	X_PBS_	X_PHA_
PLA100	-	-	176.94	47.67	-	50.88
PLA80PBS20	113.76	11.14	175.58	37.80	50.50	50.43
PLA60 PBS40	113.60	20.36	175.09	29.97	46.14	53.31
PLA40 PBS60	113.69	32.00	175.26	17.42	48.35	46.48
PLA20 PBS80	114.07	46.42	174.38	6.99	52.61	37.29
PBS100	114.39	59.17	-	-	53.64	-

**Table 4:** Transition temperature and crystallinity of PHA/PBS blends.

Formulations	T_m1_	$\Delta H_{m1}^{0}$	T_m2_	$\Delta H_{m2}^{0}$	XPBS	XPHA
PHA100	-	-	143.72	46.28	-	31.63
PHA80PBS20	113.25	14.71	142.85	30.23	66.68	25.83
PHA60PBS40	113.70	36.63	142.75	12.78	83.02	14.56
PHA40PBS60	113.67	37.37	142.23	12.77	56.47	21.82
PHA20PBS80	114.33	42.05	143.14	1.58	47.65	5.40
PBS100	114.67	59.17	-	-	53.64	-

**Table 5:** Transition temperature and crystallinity of TPS/PBS blends.

Formulations	T_m1_	$\Delta H_{m1}^{0}$	T_m2_	$\Delta H_{m2}^{0}$	X_PBS_	X_TPS_
TPS100	-	-	169.22	24.11	-	-
TPS80 PBS20	113.32	12.53	169.32	18.81	56.80	-
TPS60PBS40	113.46	21.74	169.12	12.67	49.27	-
TPS40PBS60	114.62	43.22	169.24	10.10	65.31	-
TPS20PBS80	114.82	54.56	168.55	4.47	61.83	-
PBS100	114.10	59.17	-	-	53.64	-

**Table 6:** Tensile test and Charpy tests results of PLA/PBS blends.

	Material	E_t_ (MPa)	σ_m_ (MPa)	ε_b_ (%)	Charpy Impact (kJ/m^2^)
PLA/PBS blends	PLA100	2940 ± 26	60.4 ± 2.4	2.50 ± 0.11	1.8 ± 0.2
	PLA80/PBS20	1806 ± 96	56.2 ± 0.6	4.8 ± 1.5	1.2 ± 0.3
	PLA60/PBS40	1510 ± 132	46.4 ± 2.4	8.4 ± 1.9	1.4 ± 0.1
	PBS100	607 ± 18	34.6 ± 0.6	120 ± 20	4.9 ± 1.2
TPS/PBS blends	TPS100	1690 ± 35	34.2 ± 0.3	26 ± 3	6.2 ± 0.8
	TPS80/PBS20	1328 ± 106	30.9 ± 1.3	34 ± 8	4.6 ± 0.8
	TPS60/PBS40	1182 ± 93	29,6 ± 1.6	207 ± 30	4.6 ± 1.0
	PBS100	607 ± 18	34.6 ± 0.6	120 ± 20	4.9 ± 1.2
PHA/PBS blends	PHA100	1090 ± 73	25.6 ± 1.1	7.5 ± 0.8	1.7 ± 0.1
	PHA80/PBS20	907 ± 58	27.7 ± 0.4	19 ± 5	1.9 ± 0.2
	PHA60/PBS40	766 ± 61	29.5 ± 0.2	42 ± 8	2.3 ± 0.1
	PBS100	607 ± 18	34.6 ± 0.6	120 ± 20	4.9 ± 1.2

**Table 7:** Flexural test results of PLA/PBS blends.

	Material	E_f_ (MPa)	σ_fm_ (MPa)	ε_fb_ (%)
PLA/PBS blends	PLA100	3000 ± 58	85 ± 5	2.9 ± 0.2
	PLA80-PBS20	2710 ± 68	87 ± 3	4.3 ± 0.3
	PLA60-PBS40	1990 ± 51	78 ± 3	4.80 ± 0.13
	PBS100	615 ± 20	39.5 ± 1.2	9.9 ± 0.2
TPS/PBS blends	TPS100	1670 ± 65	55.1 ± 0.7	4.30 ± 0.12
	TPS80-PBS20	1510 ± 41	55.7 ± 0.6	5.4 ± 0.3
	TPS60-PBS40	422 ± 33	21.9 ± 0.5	9.79 ± 0.05
	PBS100	615 ± 20	39.5 ± 1.2	9.9 ± 0.2
PHA/PBS blends	PHA100	1420 ± 13	45.7 ± 0.7	7.20 ± 0.24
	PHA80-PBS20	1410 ± 79	49.3 ± 0.3	7.6 ± 0.4
	PHA60-PBS40	1230 ± 50	47.7 ± 0.6	8.3 ± 0.4
	PBS100	615 ± 20	39.5 ± 1.2	9.9 ± 0.2

**Table 8:** Tensile test results of PLA/EVA additive blends.

Material	E_t_ (MPa)	σ_m_ (MPa)	ε_b_ (%)
PLA100	2940 ± 26	60.4 ± 2.4	2.50 ± 0.11
PLA95/Ad5	2292 ± 203	39.0 ± 4.0	11.8 ± 2.80
PLA80/Ad10	2038 ± 210	38.1 ± 2.1	9.40 ± 1.90
PLA85/Ad15	1852 ± 50	25.6 ± 8.3	12.7 ± 2.1
PLA80/Ad20	1686 ± 23	21.2 ± 5.1	26 ± 3.0

## Data Availability

Not applicable.
